# Sex differences in the winter activity of desert hedgehogs (*Paraechinus aethiopicus*) in a resource-rich habitat in Qatar

**DOI:** 10.1038/s41598-022-15383-4

**Published:** 2022-07-01

**Authors:** Carly E. Pettett, Rosie D. Salazar, Afra Al-Hajri, Hayat Al-Jabiri, David W. Macdonald, Nobuyuki Yamaguchi

**Affiliations:** 1 WildCRU, Department of Zoology, University of Oxford, The Recanati-Kaplan Centre, Tubney House, Abingdon Road, Tubney, Abingdon, OX13 5QL UK; 2 Department of Biological and Environmental Sciences, College of Arts and Sciences, Qatar University, PO Box 2713, Doha, Qatar; 3 Institute of Tropical Biodiversity and Sustainable Development, University of Malaysia Terengganu, 21030 Kuala Nerus, Malaysia

**Keywords:** Behavioural ecology, Animal behaviour

## Abstract

Hedgehogs’ wide distribution and breadth of habitat use means they are a good model taxon for investigating behavioural responses to winter conditions, such as low temperatures and resource availability. We investigated the over-winter behaviour of desert hedgehogs (*Paraechinus aethiopicus*) in Qatar by radio-tracking 20 individuals and monitoring the body mass of 31 hedgehogs. Females spent more nights (38.63% of nights tracked) inactive than males (12.6%) and had lower monthly activity levels. The mean temperature on nights where hedgehogs were inactive was 14.9 °C compared with 17.0 °C when hedgehogs were active. By December, females lost a higher percentage of their November body mass than did males, but by February males had lost a higher percentage than females. We conclude that these sex differences in behaviour are a result of differing reproductive strategies with males becoming more active early in spring to search for mates, whereas female hedgehogs conserve energy for producing and raising young and avoid harassment by males. The winter activity of males may be facilitated by the resource-rich environment created by humans at this study site, and basking behaviour. This study highlights intraspecific and interspecific variation in behavioural strategies/tactics in response to winter conditions.

## Introduction

Over winter small mammals face the challenge of maintaining endothermy when ambient temperatures are lower than optimum core body temperature, thus making the energetic cost of thermoregulation high^1,2^. Additionally resource availability may fall over winter resulting in further energetic constraints. To address this challenge, individuals may display behavioural plasticity to respond quickly to changing ambient conditions^3^. Traditionally behavioural ecologists have split behavioural choices into strategy, a stable inherited trait in the evolutionary temporal scale, or tactic, a trait which may vary in response to environmental factors and may include learned behaviour^4^. However, the situation may be more nuanced than this with some behavioural responses to varying environmental conditions being a combination of inherited strategies, tactics and learnt behaviour^4,5^. This complex interaction can lead to intra-specific variation in behavioural responses to environmental stimuli^4^, and here we investigate how and why individuals may vary in their response to winter conditions in the northern hemisphere.

Behavioural responses of small mammals to winter conditions may include displaying shorter bouts of activity when ambient temperatures are lower^6,7^ or during cold temperature extremes^8^, spending more time in a nest or burrow^8,9^ or changing timings of peak activity^7,10^. Nest selection may also change seasonally according to thermoregulatory needs^6^. In some species individuals may use bouts of torpor, i.e. lower their body temperature and metabolic rate to conserve energy^11^. Falling temperature has frequently been observed to trigger the onset of bouts of torpor over winter^12–14^, and this is seen in warmer climates as well as temperate conditions^11,14^. A further behavioural adaptation to dealing with temperature regulation in small mammals is basking^15–18^, which reduces the cost of thermoregulation and/or re-warming following torpor^17^. Torpor is not without its costs, which may include oxidative stress, reduced immunity, reduced memory function and susceptibility to predation^19,20^. Therefore a small mammal’s strategy over winter must be a balance between the detrimental effects of reduced activity or torpor and the conservation of energy, in order to minimise body mass loss and reduction in condition^19^. Changes in behaviour must also minimise predation^6^ and competition^10^.

Resource availability, such as food caches and nearby rich resources may influence overwinter behaviour, including expression of daily torpor, with bouts of torpor increasing when resources are scarce^21,22^. Body mass, i.e. stored reserves, at the start of winter may also affect torpor expression and activity^19^, with higher body mass resulting in less time spent in torpor^23^. Conversely, animals may range further when resources are dispersed^24^. Body mass/condition may also impact post-winter activity in the spring, and this may be sex specific as shown in the little brown bat (*Myotis lucifugus*) where females in good condition emerge from hibernation earlier than other females and male emergence is not influenced by body condition^25^.

Previous studies have reported intraspecific differences in winter behaviour related to sex^26–30^, or related to a combination of both sex and resources^21,25,31^. Male Anatolian ground squirrels (*Spermophilus xanthoprymnus*), Richardson ground squirrels (*Urocitellus richardsonii*) and thirteen-lined ground squirrels (*Ictidomys tridecemlineatus*) spend more time active than females through either having shorter bouts of torpor and more frequent arousals, or beginning the first torpor later and ending the last torpor bout earlier than females^12,13,32^. Similarly, adult male golden mantled ground squirrels (*Callospermophilus lateralis*) remain more active than females and juvenile males over winter by entering hibernation later and emerging earlier, suggesting an influence of both sex and reproductive status^29^.

Hedgehogs are a good model taxon for investigating factors influencing small mammal behaviour over winter because of the breadth of climatic niches and habitats they span^33^. There are 14 species of spiny hedgehogs (Erinaceinae) covering a range of habitats from temperate Western Europe to the deserts of Africa and the Middle East^34,35^. All of the spiny species of hedgehog are thought to enter torpor to some degree^34^, but behaviour varies over winter and therefore we can make comparisons of behavioural strategies/tactics under a variety of conditions. Over winter activity patterns vary both between hedgehog species and within the same species occupying differing climatic conditions^34,36^. Populations in colder climates tend to have a long period of hibernation; European hedgehogs (*Erinaceus europaeus*) in Ireland emerged from hibernation earlier in the spring^37^ than in colder parts of this species range such as in the UK, Finland, and Denmark^37–41^. In some milder regions not all individuals or species may hibernate^34,36^ and activity bouts appear to be longer in species occupying warmer arid environments such as in *Hemiechinus auritus*^14^. There is also further intraspecific variation in winter activity, as in the other small mammal species discussed above, including variation as a result of body condition^42,43^, resource availability^44,45^, sex^34,41^, temperature^46–48^ and photoperiod^47^.

In this study, we investigate the behavioural strategies of free-ranging male and female desert hedgehogs (*Paraechinus aethiopicus*) over the north hemisphere winter in Qatar, where the environmental conditions may facilitate their survival in cooler months without entering torpor for long periods. This species is distributed across North Africa, the Middle East and the Arabian Peninsula^34,35^ and is the only native hedgehog in Qatar^49^. Like its European counterparts, the species is nocturnal^50^. Studies on the habitat use and home range of the species in Qatar have found the species selects irrigated farms and human-influenced habitats^51,52^. The species displays short bouts of torpor during winter^33,50^, and breeding begins shortly after the winter period in February^53,54^. Like other hedgehogs^34^, the species displays a promiscuous mating system with males of the species having larger home ranges than females, purportedly to maximise mating opportunities^51,52^.

We studied free-ranging desert hedgehogs in an approximately 15 km^2^ arid area of Qatar containing isolated, and regularly irrigated, farms. Based on the activity data collected by radio-tracking in the field, we investigate factors affecting nightly activity levels over the winter period including temperature, sex and body mass. We also examine factors affecting body mass changes of hedgehogs over winter. We hypothesise that:

1. The resource rich habitat and relatively mild winter climate at this site will result in hedgehogs remaining active to some degree over the winter period, however;
2. Activity of both sexes will decrease over winter as males no longer need to range to look for mates and conserving energy in the face of colder temperatures becomes of principal importance;
3. Hedgehogs which lower their activity levels overwinter will conserve more energy and lose less body mass over the winter period;
4. Hedgehogs with a higher body mass will remain more active over winter;
5. Temperature will be a key predictor of activity levels over winter

## Results

We obtained activity data for 20 individuals. We tracked 10 of these (five females and five males) between November 2010 to February in 2011 and 11 (six females and five males) between November 2011 to February 2012. In 2011–2012, one female was the same individual as in 2010–2011; the others were all new individuals. No hedgehog was completely inactive over winter; instead hedgehogs had periods of inactivity in the nest, with a range of 0–56.82% of nights tracked spent completely inactive and in the nest (Table 1). Both male and female hedgehogs began bouts of inactivity from late November (Table 1). The last time an individual was inactive all night during the winter was variable. The last recorded day for males in the study ranged from 20th November and 1st January and for females it was between 15th January and 9th February, although one female was still inactive and in the nest when last tracked on 25th February (Table 1).

**Table 1:** Periods of inactivity in the nest in 20 desert hedgehogs radio-tracked overwinter in 2010–2011 and 2011–2012.

ID	Year	Sex	Date of first inactivity*	Date ended†	No. nights tracked	No. nights inactive	% nights inactive
20	2010–2011	F	23/12/2010	09/02/2011	36	8	22.22
44	2010–2011	F	–	–	3	0	NA
53	2010–2011	F	–	–	19	0	0.00
70	2010–2011	F	22/11/2010	15/01/2011	16	8	50.00
71	2010–2011	F	19/11/2010	15/01/2011	27	13	48.15
76	2010–2011	F	21/12/2010	16/01/2011	26	10	38.46
21	2010–2011	M	19/11/2010	20/11/2010	36	1	2.78
24	2010–2011	M	21/11/2010	25/11/2010	29	2	6.90
45	2010–2011	M	18/11/2010	25/11/2010	25	5	20.00
60	2010–2011	M	19/11/2010	23/12/2010	26	6	23.08
65	2010–2011	M	20/11/2010	21/11/2010	30	1	3.33
48	2011–2012	F	10/11/2011	06/01/2012	42	15	35.71
70	2011–2012	F	25/11/2011	NA	31	14	45.16
115	2011–2012	F	24/11/2011	Ongoing	44	25	56.82
116	2011–2012	F	25/11/2011	25/01/2012	48	18	37.50
124	2011–2012	F	23/11/2011	25/01/2012	44	23	52.27
18	2011–2012	M	24/11/2011	02/01/2012	49	4	8.16
49	2011–2012	M	15/12/2011	30/12/2011	46	4	8.70
51	2011–2012	M	08/11/2011	17/12/2011	49	10	20.41
81	2011–2012	M	23/11/2011	30/12/2011	46	8	17.39
97	2011–2012	M	23/11/2011	30/12/2011	46	7	15.22

“–” = Hedgehog was never inactive for a full night, “NA” = not enough data (the animal was last successfully radio-tracked on 07 January 2012 before the tag fell off) “Ongoing” = Hedgehog still inactive at end of tracking period (25 February 2012).

*One or more nights inactive and in the nest.

^†^Last night of inactivity during the tracking period.

**Figure 1:**
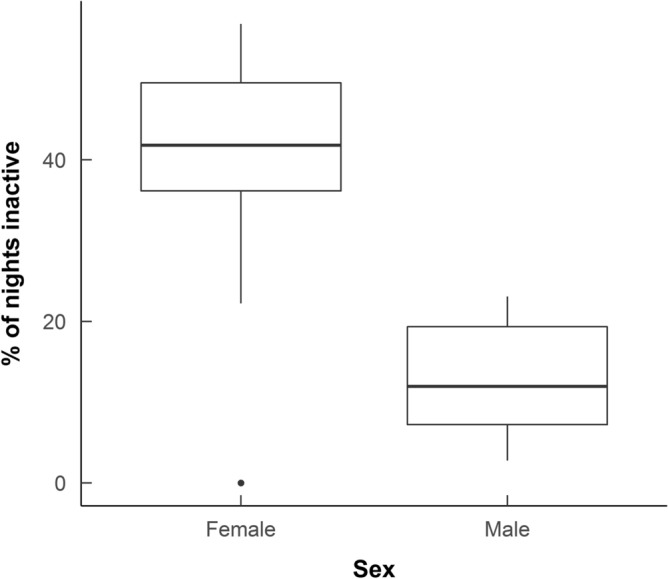
The median percentage of nights spent inactive and in the nest for male and female desert hedgehogs over winter in Qatar. The percentage was calculated by dividing the number of nights inactive divided by the total number of nights tracked over winter. Data includes 20 individuals, radio-tracked from November 2010 to February in 2011 and November 2011 to February 2012. Lower and upper hinges show the interquartile range. Whiskers show values within 1.5 times the interquartile range, dots are outliers to this.

As well as a longer period over winter where hedgehogs stayed in the nest, females spent more nights totally inactive than males (38.63 ± 5.32% (mean ± standard error) of nights tracked for females versus 12.6 ± 2.37% for males, *χ*^2^ = 73.103, p < 0.0001, df = 1, Fig. 1, Supplementary Table S1). There was no effect of starting body mass in November on the percentage of nights a hedgehog spent inactive (Supplementary Table S1). Hedgehogs tracked in 2010–2011 spent 21.49 ± 5.94% nights inactive compared with 29.73 ± 5.71% nights in 2011–2012. This annual difference was near significant in our model (*χ*^2^ = 3.427, p = 0.064, df = 1, Supplementary Table S1).

**Figure 2:**
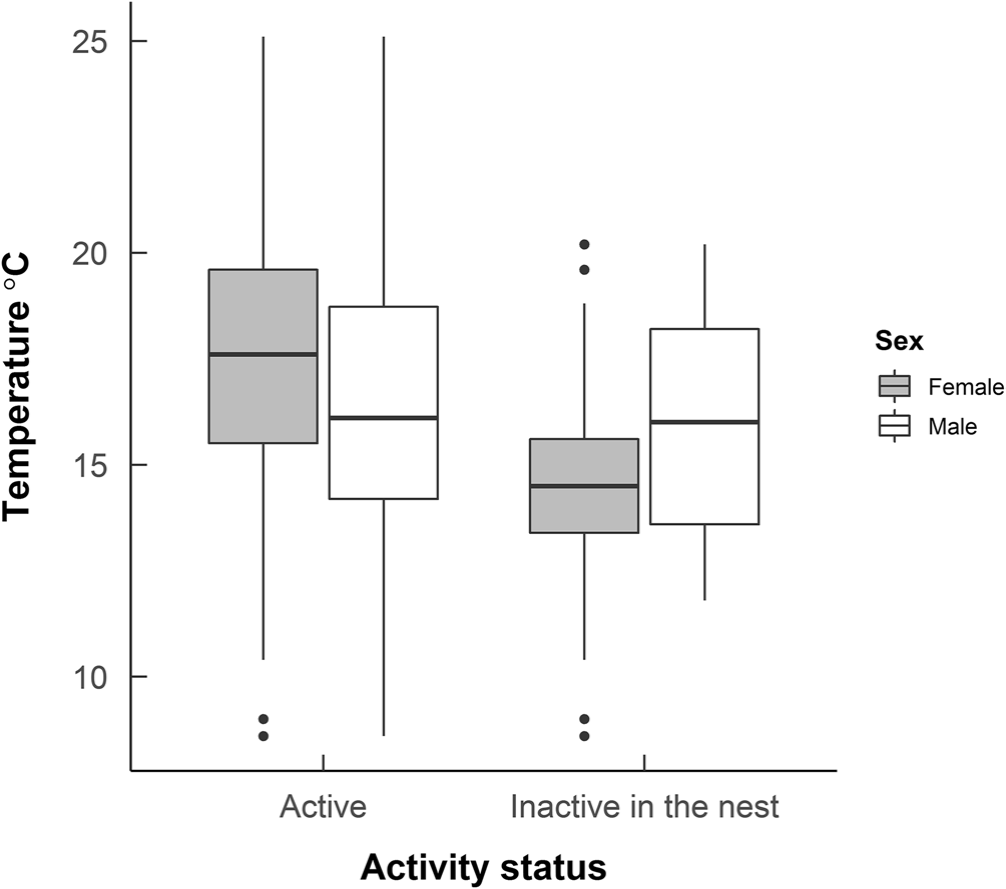
The mean nightly temperature on nights where male and female hedgehogs spent all night inactive and in the nest, compared with the mean nightly temperature when hedgehogs were active. Data includes 20 individuals, radio-tracked in Qatar from November 2010 to February in 2011 and November 2011 to February 2012. Lower and upper hinges show the interquartile range. Whiskers show values within 1.5 times the interquartile range, dots are outliers to this.

**Figure 3:**
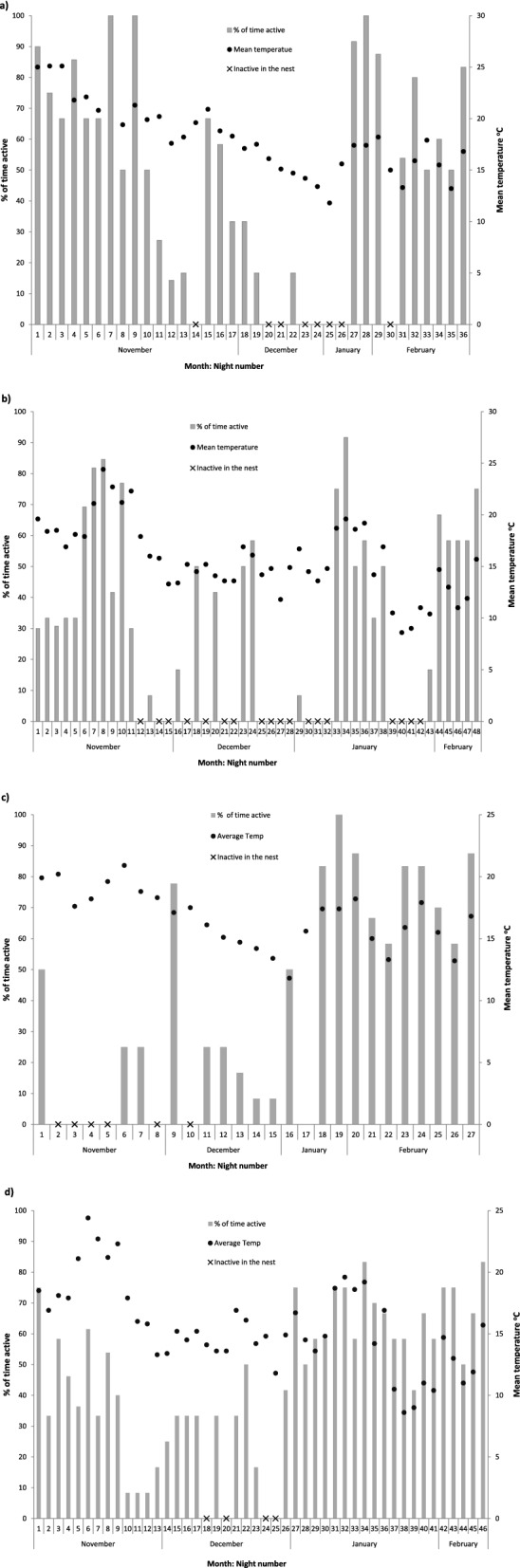
Activity data from four desert hedgehogs tracked over winter in Qatar; (**a**) hedgehog number 20 (female) tracked in 2010–2011, and hedgehog 116 (female) tracked in 2011–2012, (**c**) hedgehog number 60 (male) tracked in 2010–2011, and d) hedgehog 49 (male) tracked in 2011–2012. Grey bars show the percentage of radio-tracking fixes each hedgehog was recorded as active and outside of the nest on a given night, measured by radio-tracking signal and activity sensors. Black dots indicate the mean temperature that night. Black crosses indicate where hedgehogs were inactive and did not leave the nest all night. No bar indicates no tracking data for that night.

There was also a statistically significant effect of sex on whether or not a hedgehog was recorded as inactive on a particular night (*χ*^2^ = 75.957, p < 0.0001, df = 1, Supplementary Table S2). Temperature was a statistically significant predictor of whether a hedgehog spent the night inactive and in the nest or active and out in the open (*χ*^2^ = 54.830, p < 0.0001, df = 1, Supplementary Table S2, Figs. 2, 3). The mean temperature on nights where hedgehogs were inactive was 14.9 ± 0.2  °C (mean ± standard error) compared with 17.0 ± 0.2  °C when hedgehogs were active. The effect of temperature appears to be more pronounced in females and the interaction between sex and temperature on activity status was statistically significant (*χ*^2^ = 28.363, p < 0.0001, df = 1, Supplementary Table S2, Fig. 2).

**Figure 4:**
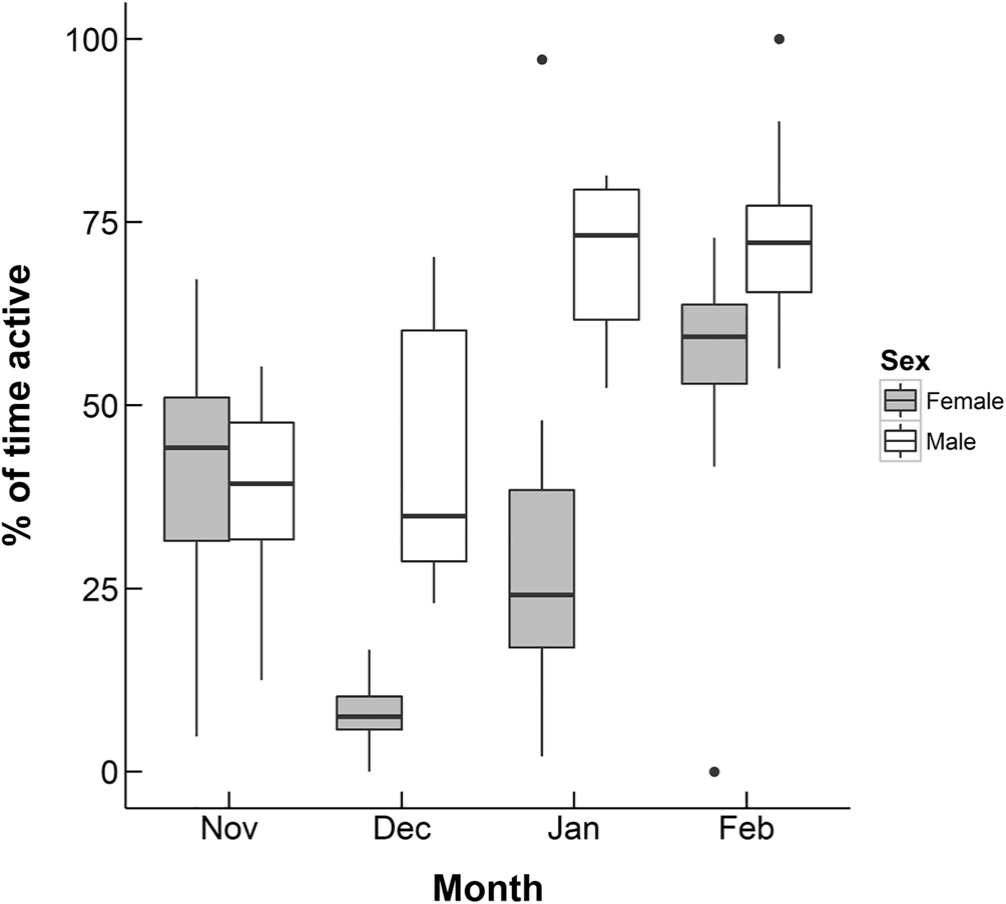
The percentage of radio-tracking fixes male and female desert hedgehogs were recorded as active and outside of the nest over winter, measured by radio-tracking signal. Data includes 20 individuals, radio-tracked in Qatar from November 2010 to February in 2011 and November 2011 to February 2012. Lower and upper hinges show the interquartile range. Whiskers show values within 1.5 times the interquartile range, dots are outliers to this.

In order to look at overall patterns in activity over winter, short of complete inactivity at night, we looked at an individual’s activity levels over each month as a whole. We found a statistically significant effect of sex on activity levels, with males having higher activity levels than females over winter (*χ*^2^ = 20.018, p < 0.0001, df = 1, Supplementary Table S3, Fig. 4). There was a significant effect of month with February having the highest activity levels for both sexes (*χ*^2^ = 369.937, p < 0.0001, df = 3, Supplementary Table S3, Fig. 4). There was also a significant interaction between sex and month. Males and females had similar activity levels in November but males were significantly more active in December and January, and then by February females increased their activity but males were still significantly more active (*χ*^2^ = 283.440, p < 0.0001, df = 3, Supplementary Table S3 and S3b, Fig. 4). There was no effect of temperature on activity in this model (*χ*^2^ = 1.132, p = 0.287, df = 1, Supplementary Table S3). For further detailed figures on individuals’ activity over the tracking period see Supplementary Information Figures S1 and S2.

We obtained 982 measures of body mass from 133 adult hedgehogs throughout 2 years of the study. The effect of month on body mass was statically significant (F_11,499_ = 23.755, p < 0.0001, Supplementary Table S4). As expected, both sexes gained weight in the autumn before the winter period and lost weight over winter (Supplementary Figure S3). There was also an effect of sex, which was statistically significant (F_1,136_ = 4.164, p = 0.043, Supplementary Table S4). The mean body mass of males was 440 ± 3 g (mean ± standard error) compared with 419 ± 4 g for females. Of higher statistical significance was the sex and month interaction on body mass (F_11,498_ = 8.22, p < 0.0001, Supplementary Table S4). Males were heavier than females every month apart from July (Supplementary Figure S4). Post-hoc tests revealed that this sex difference in body mass was statistically significant in the autumn and winter months of October, November, December, and January (Supplementary Table S4b). There was also a statistically significant effect of year on body mass; the mean body mass of hedgehogs in 2010–2011 was 446 ± 5 g compared with 424 ± 3 g in 2011–2012 (F_1,544_ = 9.364, p = 0.002, Supplementary Table S4).

**Figure 5:**
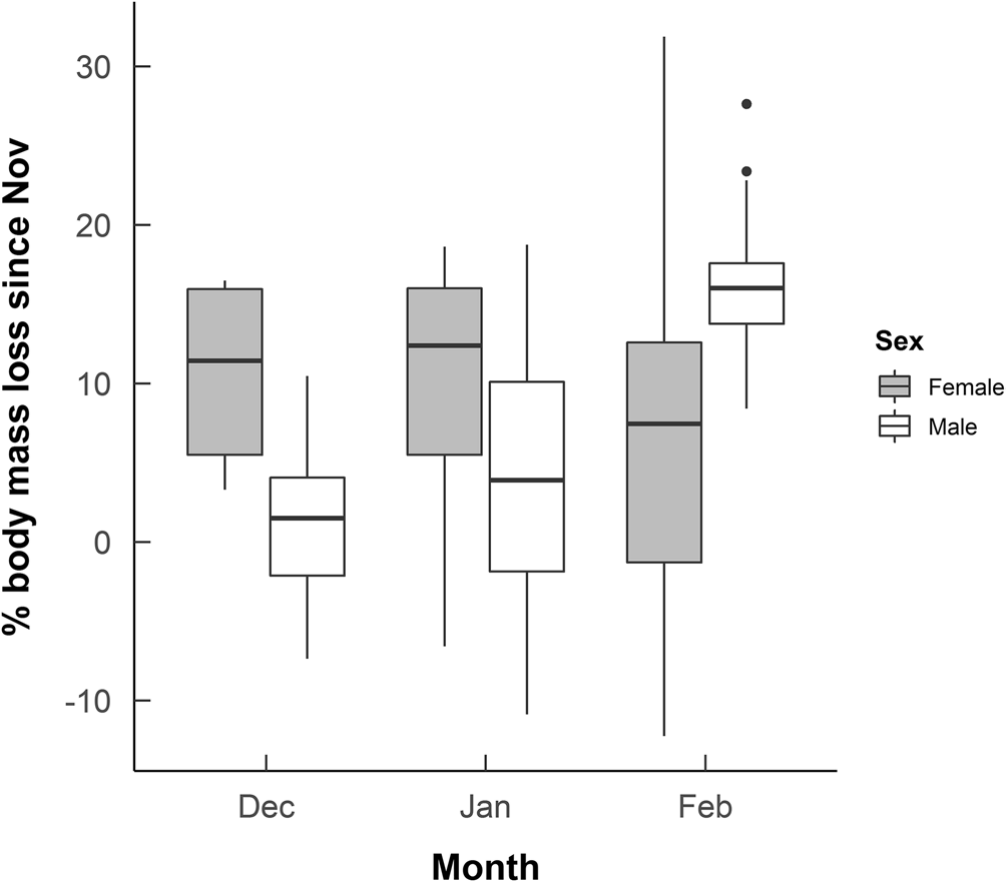
Box plot showing median percentage body mass loss between November with December, January and February for male and female desert hedgehogs in Qatar. Lower and upper hinges show the interquartile range. Whiskers show values within 1.5 times the interquartile range, dots are outliers to this. The graph shows 86 observations from 31 hedgehogs.

For the winter months (November–February), we obtained 86 measures of body mass from 31 adult hedgehogs, measured monthly over the two winter periods in the study. There was a significant effect of month on percentage body mass loss since November; hedgehogs lost progressively more weight each month (F_2,52_ = 8.864, p < 0.0001, Table 2). There was no statistically significant effect of starting mass in November (F_1,40_ = 1.560, p = 0.219, Supplementary Table S5), or year of the study on percentage body mass loss over winter (F_1,71_ = 0.033, p = 0.856, Supplementary Table S5). There was no statistically significant difference in percentage body mass loss since November between males and females (F_1,28_ = 0.508, p = 0.482, Supplementary Table S5). However, there was a significant interaction between monthly percentage body mass loss and sex (F_2,52_ = 12.871, p = 0.001, Supplementary Table S5). In December and January females had lost a higher percentage of their November body mass than males, yet by February males had lost a higher percentage of their November body mass than females (Table 2, Fig. 5). The sex difference in December and February were statistically significant but in January it was only near significance (Supplementary Table S5b).

**Table 2:** Mean monthly percentage body mass loss of male and female hedgehogs over winter in Qatar.

Sex	Month	Mean % Loss	SE
All	Dec	3.05	1.34
Female	Dec	10.53	2.68
Male	Dec	0.98	1.15
All	Jan	5.53	1.57
Female	Jan	9.38	3.00
Male	Jan	3.79	1.75
All	Feb	13.77	1.49
Female	Feb	7.77	4.04
Male	Feb	16.27	0.97

When we focused on each month separately, we found that in December females had lost a significantly higher percentage of their November body mass than did males (23 observations of body mass from 21 hedgehogs, F_1,19_ = 10.067, p = 0.005). In January, there was no statistically significant effect of sex on percentage body mass loss since November (29 observations from 26 hedgehogs, F_1,25_ = 2.418, p = 0.133). In February, males had lost a significantly higher percentage of their November body mass than females (34 observations from 31 hedgehogs, F_1,30_ = 7.22, p = 0.011). There was no significant effect of year in the study or starting body mass in November for any of the models for these three months.

We obtained 49 body mass measurements in the winter period from 18 of the hedgehogs that we also had good activity data for during the same period. We found the same patterns in monthly percentage body mass loss (Supplementary Table S6 and S6b). There were significant effects of average monthly humidity and temperature on percentage body mass loss since November (Supplementary Table S6). The higher the mean temperature that month, the greater the percentage body mass loss since November, and the higher the mean humidity the lower the percentage body mass loss in that month (Supplementary Figures S5 and S6). These measurements are not only correlated with one another but also with month and therefore results must be interpreted with caution. These results may therefore simply be another indication of the varying body mass loss each month over winter.

There was a near significant effect of activity on percentage body mass loss since November (F_1,29_ = 3.589, p = 0.068, Supplementary Table S6). The higher the activity levels, the lower the percentage body mass loss. Although this varied between the sexes, the addition of an interaction between sex and activity did not improve the fit of the model and was not statistically significant (see Supplementary Figure S7).

## Discussion

The hedgehogs in this study displayed periods of inactivity in the nest, with one animal remaining active the whole winter and others spending over 50% of nights tracked inactive, supporting our hypothesis that hedgehogs would remain active to some degree at this study site. However contrary to our hypothesis that both males and females would reduce activity to the same degree at our study site, we found sex differences in their behavioural strategies/tactics over winter. Female hedgehogs spent more time inactive than males, including more nights completely inactive and in the nest, especially in December. Males also ended these periods of inactivity sooner in the spring, with all males being completely active by early January, which is the onset of the breeding season at this study site^53^. Both males and females increased activity in February but males were still significantly more active than females, which can potentially be explained by differing reproductive strategies. Like their European counterparts, it is suggested that females of this species are more sedentary than males and focus on raising young whereas males have larger home ranges and travel to mate with multiple females^34,51^. These dissimilarities in behaviour may explain why the sexual difference in percentage body mass loss was reversed in February by which time both sexes were active yet females may be foraging to improve their body condition, whilst males are ranging further distances to mate with emerging females, which is energetically costly^55^.

Our findings indicate that, until the breeding season, hedgehogs that spend more time inactive, i.e. females, lost more of their body mass than males whose strategy was to stay active and presumably actively foraging. These findings are contrary to our hypothesis that spending more time inactive over winter would confer energy savings. If this was the case then why do females spend more time in the nest? Boyles et al.^33^ found that in the wild in Saudi Arabia, which is adjacent to Qatar, this species displays frequent and irregular bouts of torpor rarely lasting more than 24 h, unlike the longer periods of deeper seasonal hibernation seen in hedgehogs living in temperate environments. Although in this study we did not measure body temperature of the animals, it is likely that some of this time spent inactive in the nest was spent in torpor.

One potential explanation for males spending less time in the nest, i.e. in torpor, is that where energy expenditure is expected to be high on emergence, for example due to the requirement for increased mobility to find a mate or spermatogenesis, males may show a different strategy in hibernation timing and duration, emerging earlier and in better condition in the spring^23,56^. Increased levels of testosterone and testes development may prevent a more plastic response to conditions in reproductive males by inhibiting torpor^57^. For example in arctic ground squirrels (*Urocitellus parryii*), females and non-reproductive males re-enter hibernation in response to delayed snow melt, in contrast with reproductive males^28^, potentially due to high androgen levels in such males^58^. In male desert hedgehogs testosterone levels are highest in winter (December–February) followed by an increase in testes size in late winter and spring^54^, and therefore, such a high testosterone level may limit the expression of torpor in male desert hedgehogs.

Hibernating hedgehogs may use 15% of the monthly energy expenditure that an active hedgehog uses^46^, and like other small mammal species they have been shown to use torpor when resources are restricted, for example in laboratory experiments on the southern African hedgehog (*Atelerix frontalis*)^44^. In order to survive the winter period whilst spending more time active, male desert hedgehogs must therefore obtain sufficient resources. When European hedgehogs in the UK were able to access supplementary food in gardens throughout winter, they were observed more frequently^45^, suggesting that access to increased anthropogenic resources potentially caused hedgehogs to spend more time active. Throughout the Arabian Desert and Gulf Region, including Qatar, the ‘economic miracle’ of oil and natural gas extraction during the last few decades has triggered an extreme transformation of much of the formerly barren desert into urban areas, small and large-scale agricultural farms, and industrial developments^52^. This recent change in land use is expected to influence local resource availability, and desert hedgehogs in Qatar inhabiting an area with irrigated farms are significantly heavier, and have significantly smaller home ranges, than those in more natural desert habitats^52^. Pettett et al.^51^ found that hedgehogs at our study site selected rubbish dumping sites, which were resource rich and therefore this availability of food may allow males to maintain their body mass in winter, which could potentially support the idea that the resources at this site may facilitate more active behaviour over winter. In other words, what we observe in this study, as well as from Alagaili et al*.*^35^ which was conducted around farms in Saudi Arabia, could be an artefact due to the recent change of land use in the Gulf Region.

Whether artefact or not, why do females not take advantage of these resources over the winter? As well as the lower testosterone levels and a differing breeding strategy described above, we also speculate that this may be due to a further phenomenon; females may not be able to forage as efficiently as we expect due to the harassment by mate-seeking males, which are active during the winter. Observations of behaviour at the site may tentatively support this; on 11th January 2012 we observed one female being courted by two males in the early evening and a further male in the early hours of the morning. During these interactions the female was stationary (see full description in supplementary information). Studies of European hedgehogs indicate that courtship only occurs in the breeding season^59^, and although that is also usually the case for this species^50,54^, testosterone levels are high in winter^54^ and some courtship begins in January at this study site^53^. Therefore, any active females may face interruptions to foraging because of interactions with males and females may not be able to take advantage of the available resources. There is an interesting comparison here with echidnas, where males also harass females during the hibernation period, however male echidnas have been observed to disrupt the females nest and cause arousal from torpor^60^, which has not been observed at our study site. If the high resource availability at this site continues, it will be interesting to investigate whether females at our study site become as active as males during the winter, or if they are in some way limited by male activity at the site.

Temperature was a statistically significant predictor of whether a hedgehog spent the night inactive and in the nest or active and out in the open, corroborating our hypothesis that temperature would be a key factor affecting over winter activity. However, we did not find a significant effect of temperature on activity at the monthly scale, suggesting that the temperature on an individual night is a better predictor of activity than the conditions over a longer period. The effect of temperature on use of torpor/inactivity in hedgehogs has been previously shown in several species in captivity^14,44^. Desert hedgehogs also show basking behaviour which may be important for returning to euthermy after bouts of torpor^15^. This reduced energy expenditure during rewarming may possibly facilitate this species to remain more active over winter and display shorter bouts of torpor akin to other desert species such as in the long-eared hedgehog (*Hemiechinus auritus*)^14^.

We found no effect of body mass in November on the number of nights spent inactive, the opposite of the notion, and our hypothesis, that stored reserves may result in mammals remaining more active over winter^19^ or previous findings in the southern African hedgehog where heavier individuals displayed longer bouts of torpor^42^. There were some interesting findings around the year of the study. There was a near significant effect on percentage body mass loss and the number of nights inactive and a significant effect on mean body mass, with hedgehogs being a mean of just over 22 g lighter in the second year of the study and spending a higher percentage of nights inactive in the nest (8.24 more). These findings indicate potentially poorer conditions in the second year of the study and support our supposition that more time spent inactive in the nest may result in more body mass lost over winter. In fact, the mean temperature was colder over winter in the second year of the study (17.6 ± 0.2 °C in 2010–2011 compared with 15.7 ± 0.2 °C in 2011–2012, Supplementary information Figure S8). Additionally, a major cleaning operation at the rubbish mound first began in March 2011, which likely reduced the available resources for hedgehogs and could also explain these findings. Up until March 2012, the rubbish mound appeared to continue to attract hedgehogs but in March 2012 a further cleaning operation started around the rubbish mound to remove it completely. Furthermore, Abu Baker et al*.* 2017 monitored hedgehogs in the same area between 2014 and 2015, when the rubbish mound had been fully removed, and their mean body mass (418 g) was lower than in our study^52^.

There are many other reasons a small mammal may remain in the nest without entering torpor, for example, disturbance, predation risk, or poor weather conditions. At our study site, the only likely predator of hedgehogs was domestic dogs. We are unaware of an increased predation pressure on females from these dogs that would result in the sex differences in activity observed here. Although we found nightly temperature to be a predictor of inactivity, rain was a very rare phenomenon at the site and so could not be responsible for the frequent bouts of inactivity observed. We did not quantitatively measure light pollution at the site but based on observation with the naked eye, the brightest light source was a large highway just outside of the eastern edge of the study site, approximately one kilometre away from the area where most animals were radio-tracked. There were also street lights at the research station but these were not very bright. Therefore, we do not believe any unnatural light sources at the study site impacted the hedgehogs’ activity. Further study of activity in relation to body temperature, predation risk and disturbance would help to disentangle these effects.

## Conclusions

In this study, we uncovered differing behavioural strategies of male and female desert hedgehogs in Qatar over winter. Female hedgehogs lowered their activity in the winter months, potentially to conserve energy for producing and raising young in the spring. Males on the other hand spent more time active and less time in a nest. Male inactivity may be limited by intrinsic factors including the need to increase testosterone production in late winter and emerge early, ready for mating in the spring. The ability of males to remain more active over winter without losing body mass may be facilitated by the resource rich habitats at this study site, such as irrigated farms and large rubbish dumping sites, which have become available in the Gulf Region recently, and basking behaviour. It may also pay for females to spend more time in the nest over winter as they may be unable to maintain sufficient weight as foraging is interrupted by males. It appears at our study site, male and female hedgehogs used different strategies/tactics to meet the same ends, and these strategies may have come about as a result of complex interactions between climatic conditions, thermoregulatory constraints, resource availability, mating systems, and how the two sexes have adapted to these conditions.

## Methods

### Study site

**Figure 6:**
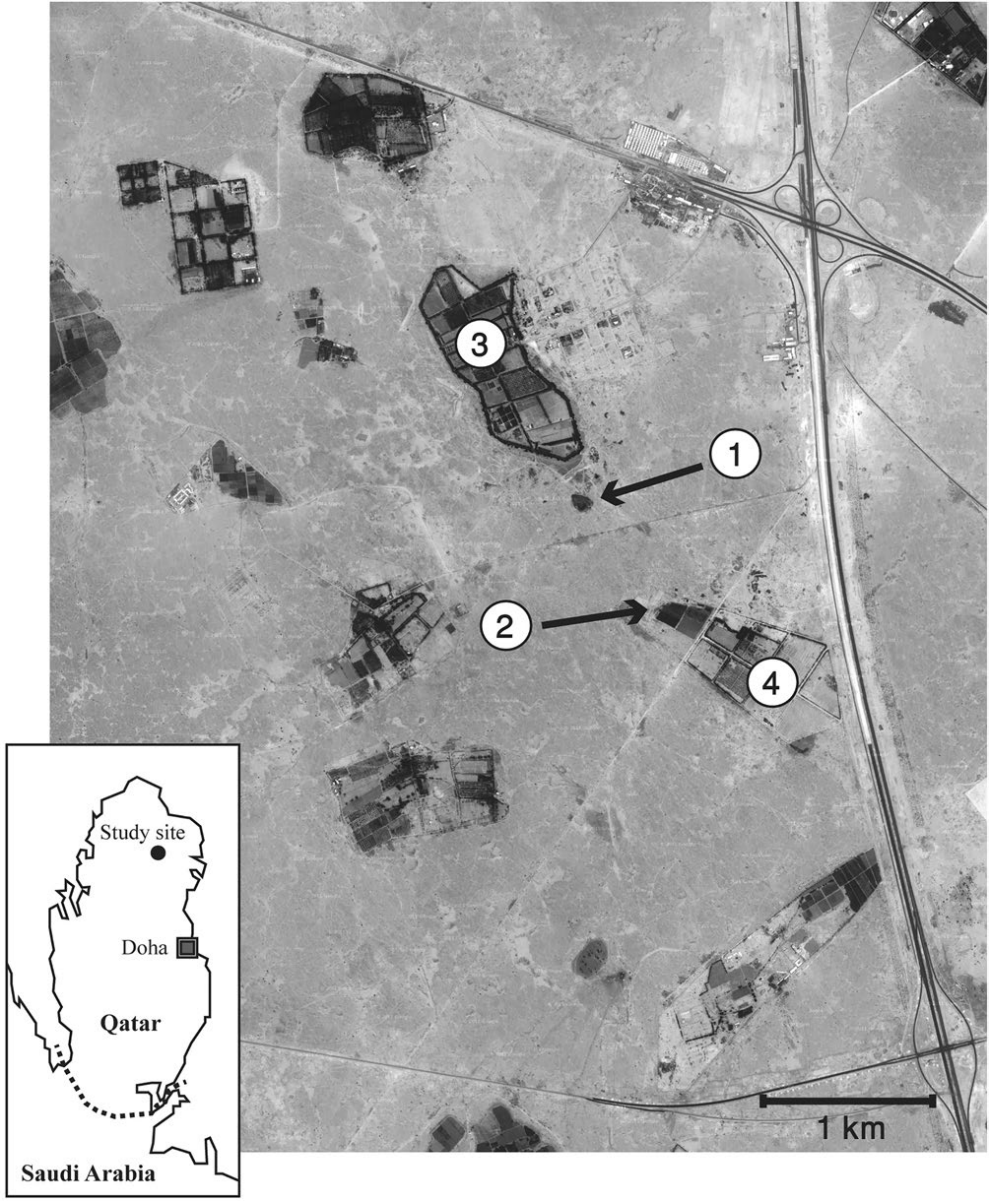
Map of the study site (GoogleEarth Image Copyright 2011 DigitalGlobe): (1) the “Rubbish Mound” where a higher concentration of hedgehogs was found throughout the year probably due to year-round availability of food resources. (2) “Municipal Farm” where permanent grass fields attracted hedgehogs. (3) Rawdat Al-Faras Research Station where street lights across the farm increased the chance of locating hedgehogs. (4) Qatar University Farm where the field station was located.

The study site consisted of approximately 15 km^2^ around Qatar University Farm (Fig. 6) in northern Qatar (2548′ N, 51° 20′ E). The area was arid (total annual precipitation is less than 100 mm), though included 11 active farms which were regularly irrigated. Ambient air temperature reaches above 50 °C in the summer, but ranged between c. 5 °C and c. 35 °C over the study period (between November and February, Supplementary Figure S8). Habitat types were characterised by farms, limited vegetation in the arid area with isolated low growing acacia, ephemeral grass emerging after rain in cooler months and anthropogenic habitats such as rubbish dumps, soil mounds and discarded building materials.

### Hedgehog capture

Hedgehogs were captured, individually identified, and examined for their sex, age class, and body mass (previously described in Abu Baker et al*.* 2017 and Pettett et al*.* 2018 and 2020^51,52,61^) between April 2010 and April 2012 during a monthly survey over four consecutive days. Body mass was measured to the nearest five grams. Special attention was paid to the rubbish mound, municipal farm, Rawdat Al-Faras Research Station and Qatar University Farm (Fig. 6). The rubbish mound and municipal farm attracted hedgehogs due to relative abundance of food resources and permanent grassland, respectively. In addition to the monthly capture survey, hedgehogs were captured opportunistically whenever they were encountered during field work, and also a special effort was made to capture radio-tagged animals at least once a month for monitoring body mass changes.

### Body mass

It has been reported that torpor occurs between December and February in captive desert hedgehogs exposed to the natural conditions in Qatar^50^, and the mating season for the desert hedgehog at this site is defined as January until July, with peak activity in February until March^53^. However, our radio-tracking data and observations suggested that wild desert hedgehogs begin to reduce activity in November (Table 2, Figs. 4, 5). Therefore, pre winter body mass measurements were taken in November and we monitored the changes in body mass of the hedgehogs throughout the winter by weighing individuals whenever they were captured, aiming for at least one measurement per month between November and February.

We calculated percentage body mass change of the same individuals between the pre-winter body mass in November and every subsequent month until February. In addition to the body mass measurements over winter we also measured body mass at capture throughout the year as a comparison with winter body mass (Supplementary Figure S3).

### Radio-tracking

In addition to the measures of body mass we also aimed to follow a subset of individuals more closely in order to monitor activity over winter. These adult animals were fitted with VHF radio-transmitters (TW51 single celled tag, 164 MHz frequency range, Biotrack Ltd., Wareham, UK), and followed between dusk and dawn using hand-held flexible three element Yagi aerials and Sika receivers (Biotrack). We made one radio-fix per hour per animal wherever possible. We were usually able to accurately locate the animal from a close distance (e.g. less than 50 m) with minimum disturbance to the animal when it was in an area with many different physical features (e.g. dense vegetation, dumping site, pile of building materials). When an animal was in an open area it appeared to run away from approaching observers, and hence the radio-tracking was carried out by following standard triangulation methodology^62^, usually within c. 200 m from the focal animals. A subset of thirteen radio-transmitters were fitted with activity sensors (ACT, Biotrack).

The focal animal’s activity level (inactive versus active) and whether the hedgehog was in a nest or out in the open was also recorded at each hourly location fix. When a transmitter was equipped with an activity sensor, we classified an animal’s activity level as either inactive (only inactive signals), or active (active signals) after listening to the signal from the transmitter for one minute. When a transmitter was not equipped with the activity sensor, it was classified as either inactive (an animal was in a nest and location was not moving) and active (an animal was out of its nest and moving locations). Nightly and monthly activity levels were summarised as hours active and outside of the nest divided by the number of hours monitored that evening.

During the radio-tracking, temperature and relative humidity were also measured to 0.1 °C (Kestrel 3000, Pocket Weather Meter, Nielsen-Kellerman, Boothwyn, USA) and summarised per evening as mean, minimum and maximum hourly temperature and humidity.

### Statistical analysis

All statistical analysis was performed in R^63^ (Table 3). To analyse factors affecting changes in body mass over winter, data was compiled with body mass loss (as percentage body mass loss from November that year) as the outcome variable (Table 3). We also ran three simpler models with three separate files, one for December, January and February (Table 3). We repeated our analysis of factors affecting monthly body mass changes over winter on the subset of hedgehogs that we obtained radio-tracking activity data for so that activity levels could be included as an explanatory variable (Table 3). In addition to body mass changes over winter we did an analysis to look at factors affecting body mass throughout the year (Table 3).

**Table 3:** The series of models used to test for patterns in hedgehog body mass changes and activity over winter in Qatar.

Model	Model type	R package	Response variable	Explanatory variables	Random factors	Model Assumptions	Notes
*Number of nights inactive*	GLM	p values: Anova function (car)^64^	Nights inactive/no.of nights tracked	Sex, year, month, body mass in November	N/A	Met	The binomial family and logit link was specified. p values were calculated using Type II Wald chisquare tests
*Predictors of inactivity*	Mixed effects- logistic regression	glmer (lme4) p values: Anova function (car)	Whether a hedgehog was inactive or active on a given night	Mean temperature that night, sex, sex*temperature	Hedgehog ID	Met	The binomial family was specified. p values were calculated using Type II Wald chisquare tests
*Activity levels over winter*	Mixed effects	glmer (lme4) p values: Anova function (car)	Total hours active in a given month/number of hours monitored	sex, month, mean temperature, month*sex	Hedgehog ID	Equal variances- met Some non-normality in residuals	The binomial family was specified. We did not include average humidity as it was correlated with mean temperature. p values were calculated using Type II Wald chisquare tests. Non-normality in residuals was vastly improved with the logit transformation
*Changes in body mass over winter*	Mixed effects	Lmer (lme4) p values: lmerTest	% body mass loss in December (since November)	Sex, year, month, body mass in November	Hedgehog ID	Met	
*Changes in body mass over winter*	Mixed effects	Lmer (lme4) p values: lmerTest	% body mass loss in January (since November)	Sex, year, month, body mass in November	Hedgehog ID	Met	
*Changes in body mass over winter*	Mixed effects	Lmer (lme4) p values: lmerTest	% body mass loss in February (since November)	Sex, year, month, body mass in November	Hedgehog ID	Met	
*Changes in body mass over winter: subset of hedgehogs with activity data*	Mixed effects	glmer (lme4) p values: Anova function (car)	% body mass loss since November	Sex, year, month, body mass in November, mean temp, mean humidity, activity levels, month*sex	Hedgehog ID	Met	Activity levels here was were the percentage of fixes a hedgehog was active and out of the nest in a given month
*Annual changes in body mass*	Mixed effects	Lmer (lme4) p values: lmerTest Post-hoc: emmeans	Hedgehog body mass	Sex, year, month, month*sex	Hedgehog ID	Met	Post-hoc tests were performed with the Kenward-Roger degrees of freedom correction

We performed three analyses on the radio-tracking data to examine activity over winter; one investigating factors affecting the number of nights a hedgehog spent inactive and in the nest, for at least one night; one investigating the factors affecting whether a hedgehog was inactive and in the nest or not on a particular night, and one looking at factors affecting activity levels each month over winter, using the percentage of radio-tracking fixes a hedgehog was active and outside of the nest (Table 3). We looked at these three measures of activity to ascertain whether there were not only patterns in the use of bouts of inactivity in the nest but also in the time spent out of the nest foraging.

### Ethical approval

The work was approved by the Qatar University Institutional Review Board (approval number: QUIRB 136-E/12). All experiments were performed in accordance with relevant guidelines and regulations including following the recommendations in the ARRIVE guidelines.

## Supplementary Information

Supplementary Information.

## Data Availability

The datasets generated and analysed during the current study are not publicly available due to being used in further work which is not yet published, but are available from the corresponding author on reasonable request.
