# Supplementary material for: Sex differences in the winter activity of desert hedgehogs (Paraechinus aethiopicus) in a resource-rich habitat in Qatar
Source: Sci Rep. 2022 Jul 1;12:11118. doi: 10.1038/s41598-022-15383-4 (PMC9249915; doi:10.1038/s41598-022-15383-4)
Supplement: Supplementary file 1 — Supplementary Information. [file 41598_2022_15383_MOESM1_ESM.pdf]

Supplementary Information

Sex differences in the winter activity of desert hedgehogs (*Paraechinus aethiopicus*) in a resource-rich habitat in Qatar

Figures

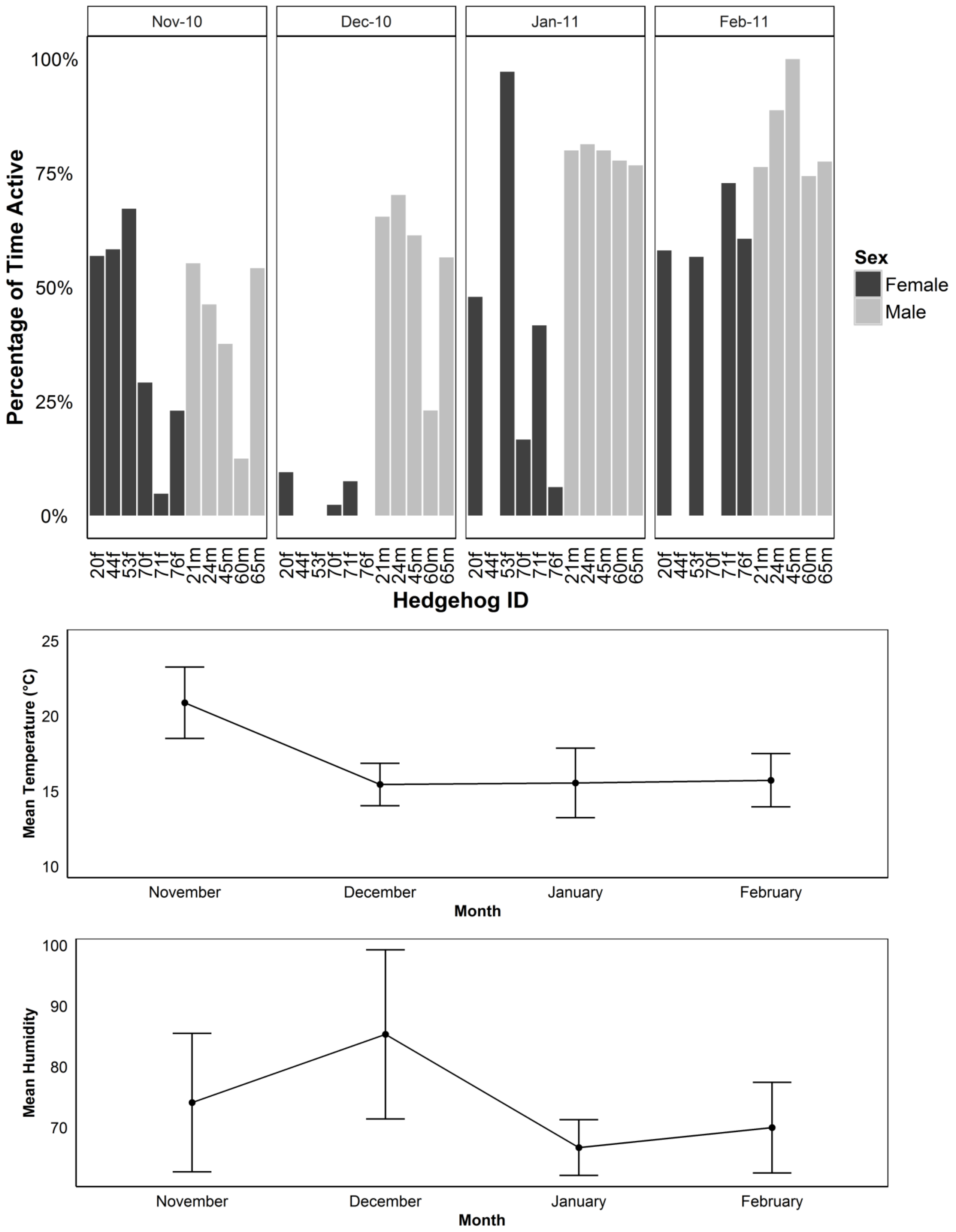

Figure S1 Plot showing the percentage of time hedgehogs were recorded as active and outside of the nest over winter in 2010-2011. The dataset includes 11 hedgehogs that were more closely followed over this period. No bar indicates no time spent active outside of the nest. Below are the mean temperature and humidity for each month of the study. Error bars indicate the standard deviation from the mean.

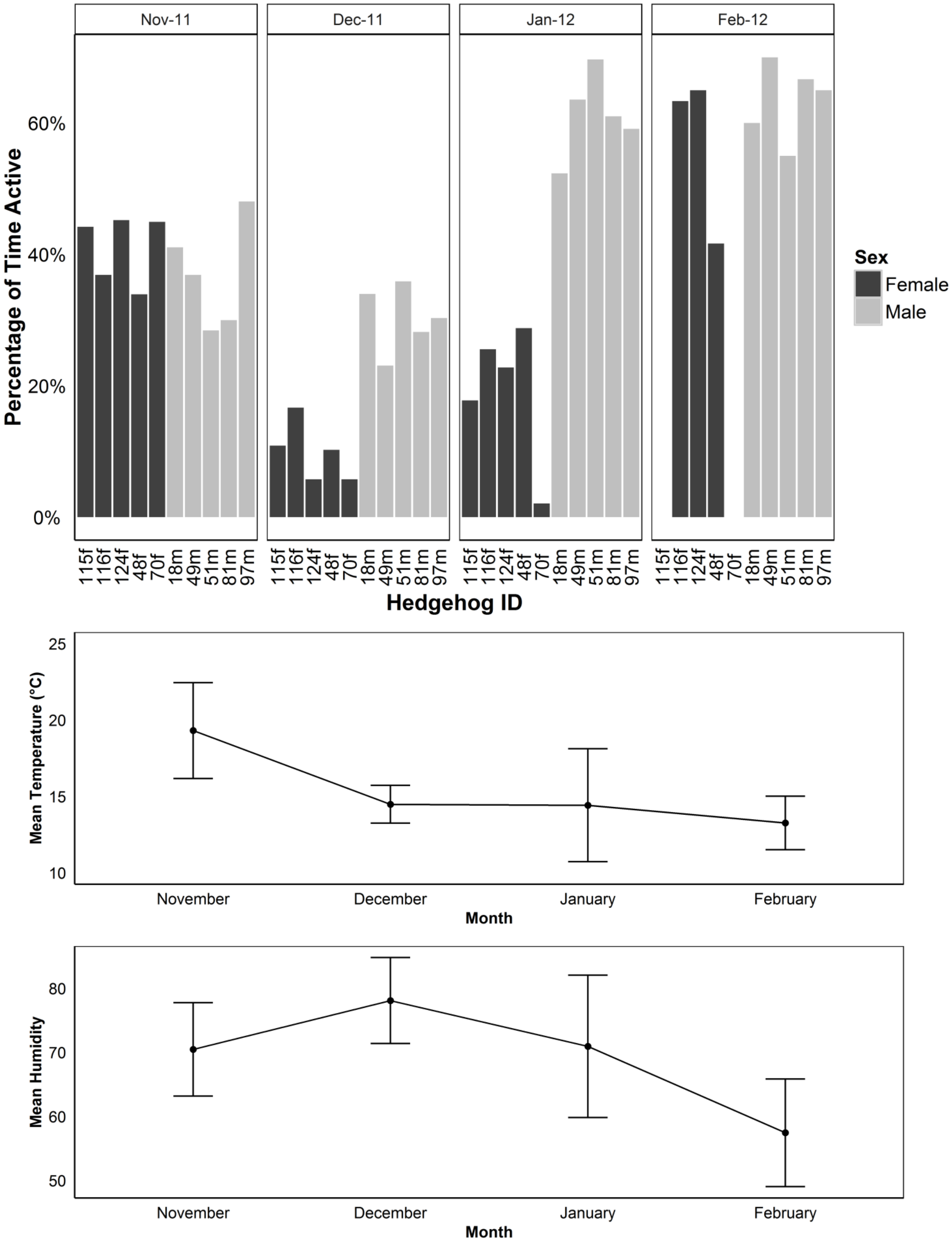

Figure S2 Plot showing the percentage of time hedgehogs were recorded as active and outside of the nest over winter 2011-2012. The dataset includes 10 hedgehogs that were more closely followed over this period. No bar indicates no time spent active outside of the nest. Below are the mean temperature and humidity for each month of the study. Error bars indicate the standard deviation from the mean.

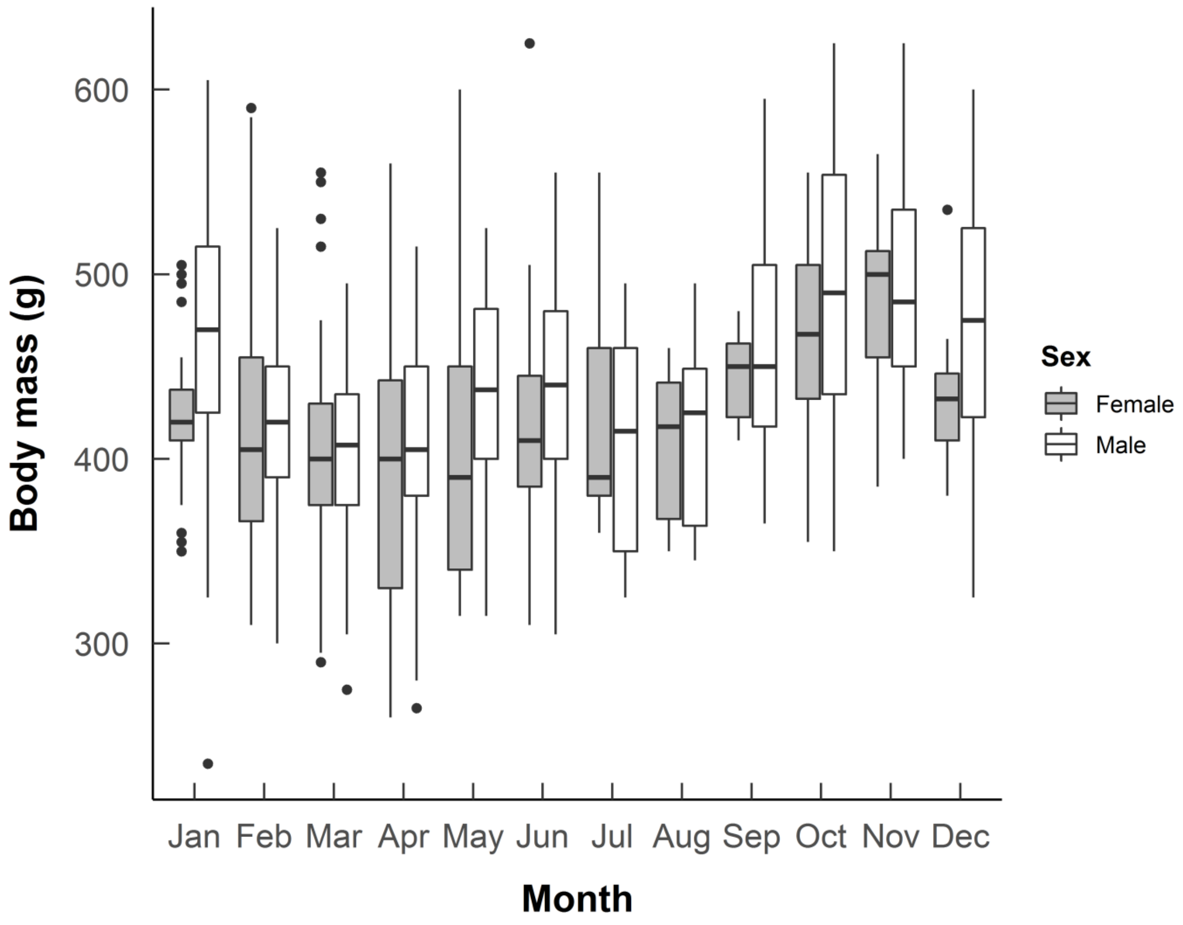

Figure S3 Box plot displaying the median monthly body mass of 133 adult male and female desert hedgehogs captured in Qatar between April 2010 and April 2012. Lower and upper hinges show the interquartile range. Whiskers show values within 1.5 times the interquartile range, dots are outliers to this.

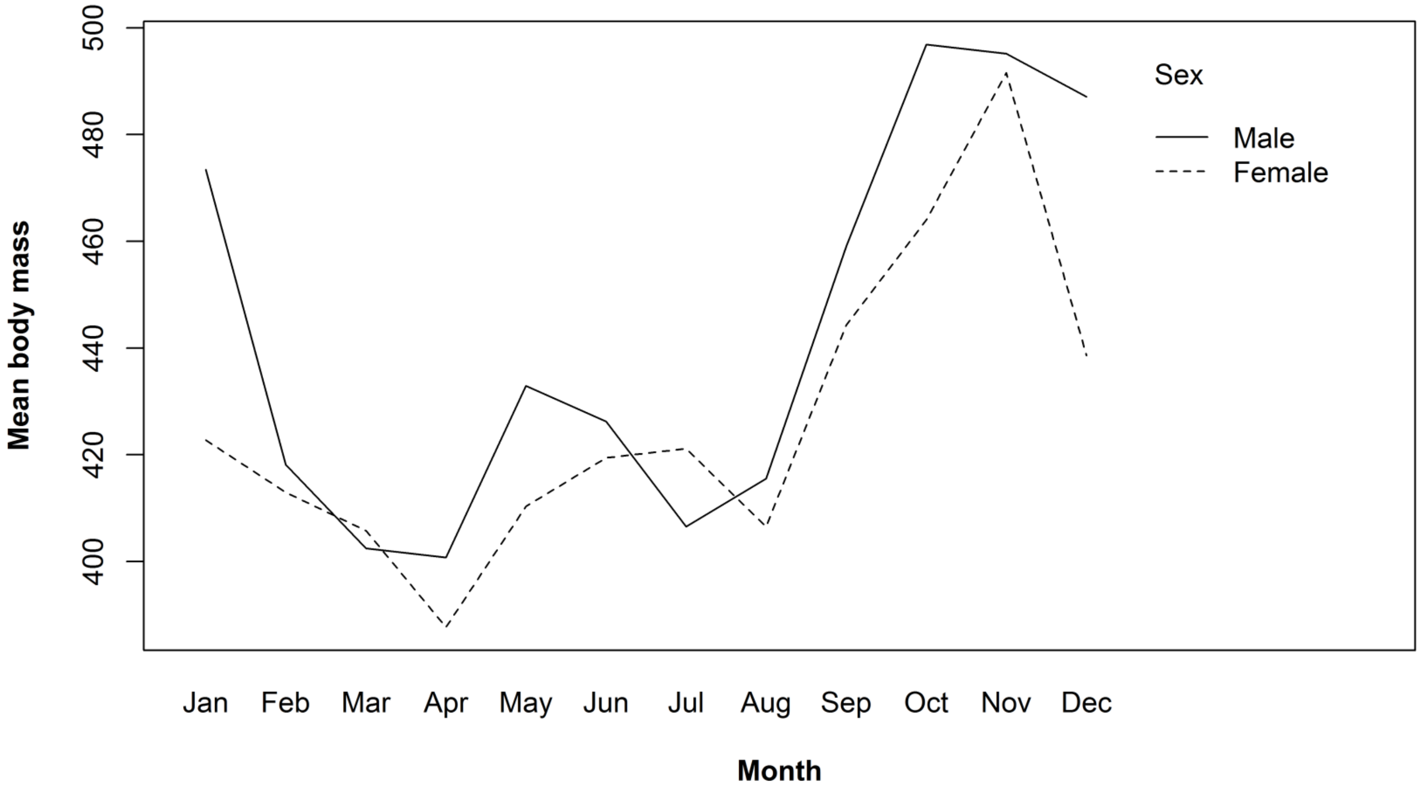

Figure S4 Interaction plot showing the effect of an interaction of month and sex on body mass of desert hedgehogs in Qatar

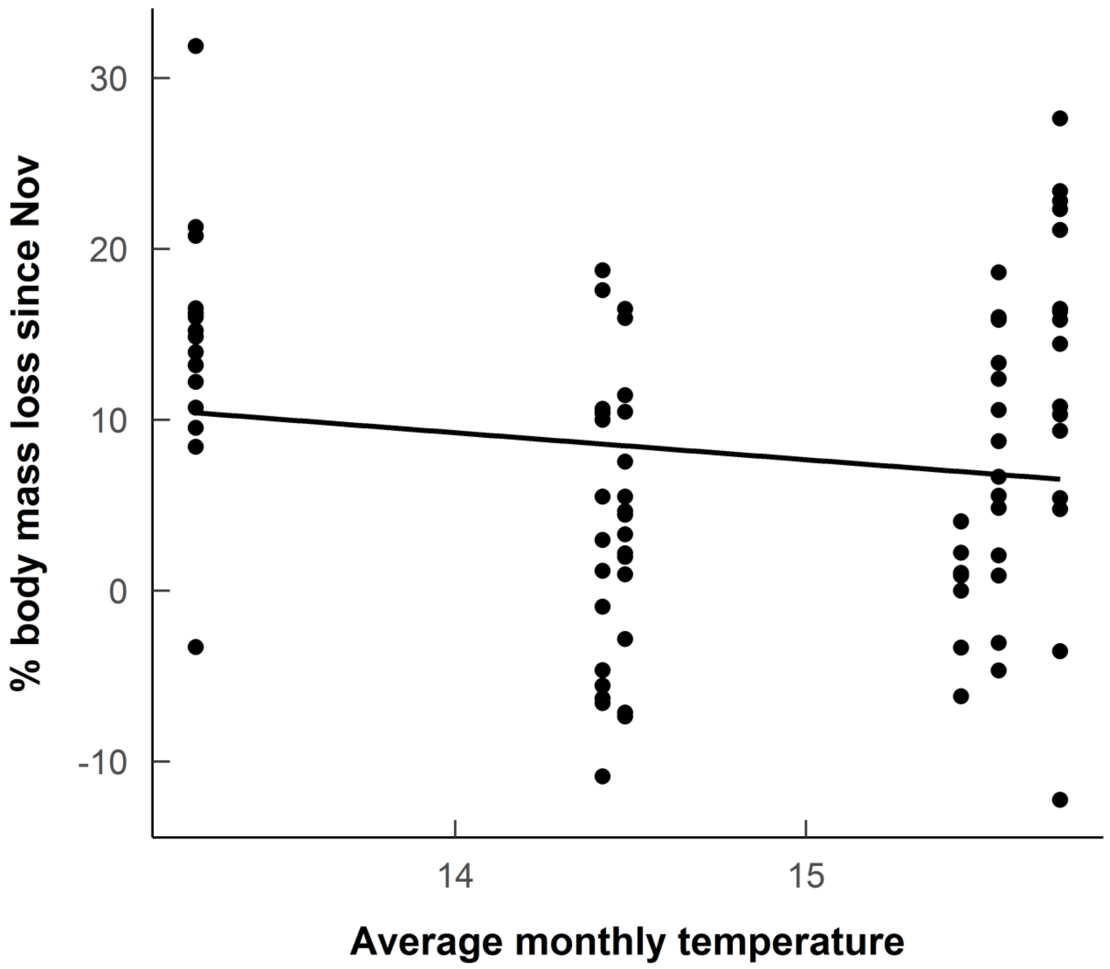

Figure S5 The relationship between the mean monthly temperature on site (degrees Celsius) and the percentage body mass loss of hedgehogs since November (measured in December, January and February)

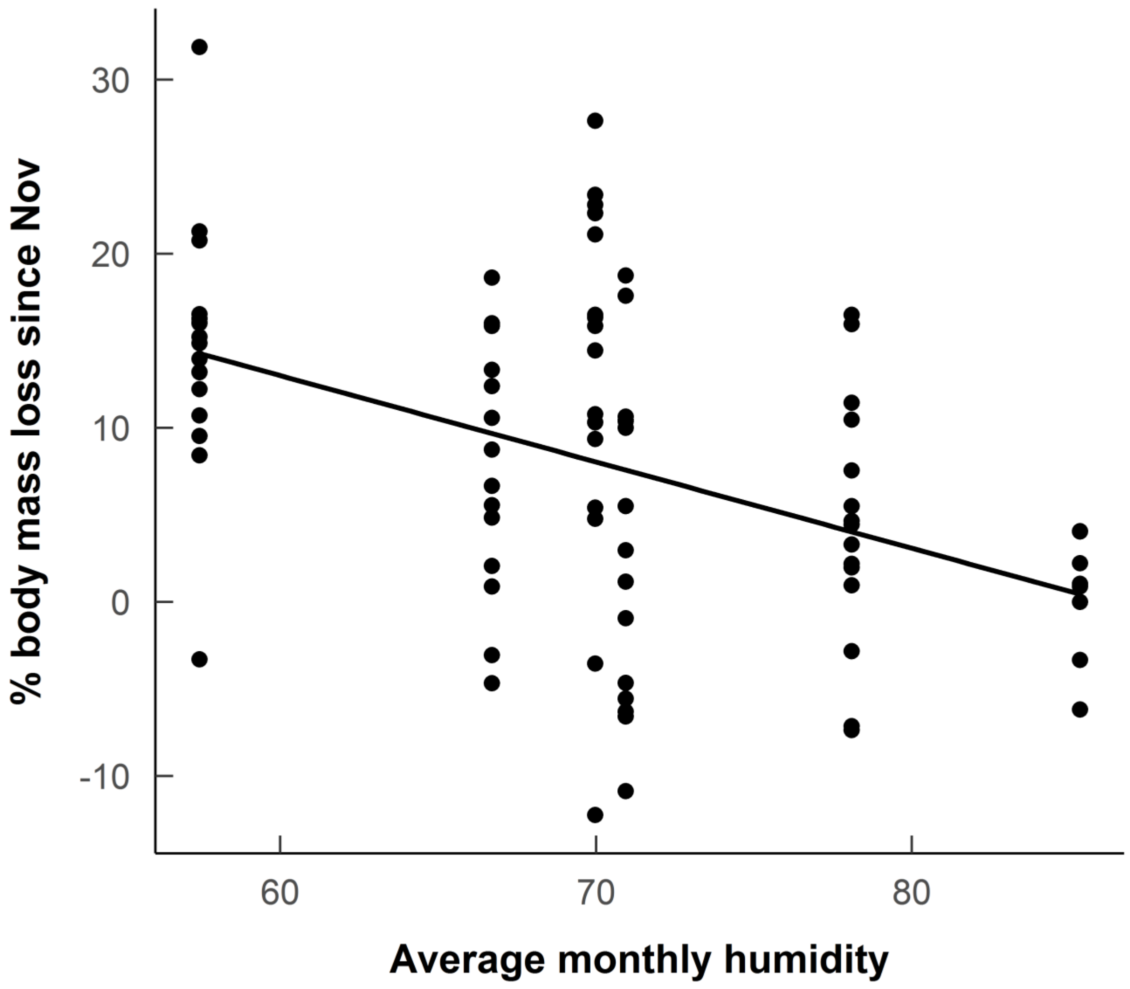

Figure S6 The relationship between the mean monthly humidity on site and the percentage body mass loss of hedgehogs since November (measured in December, January and February)

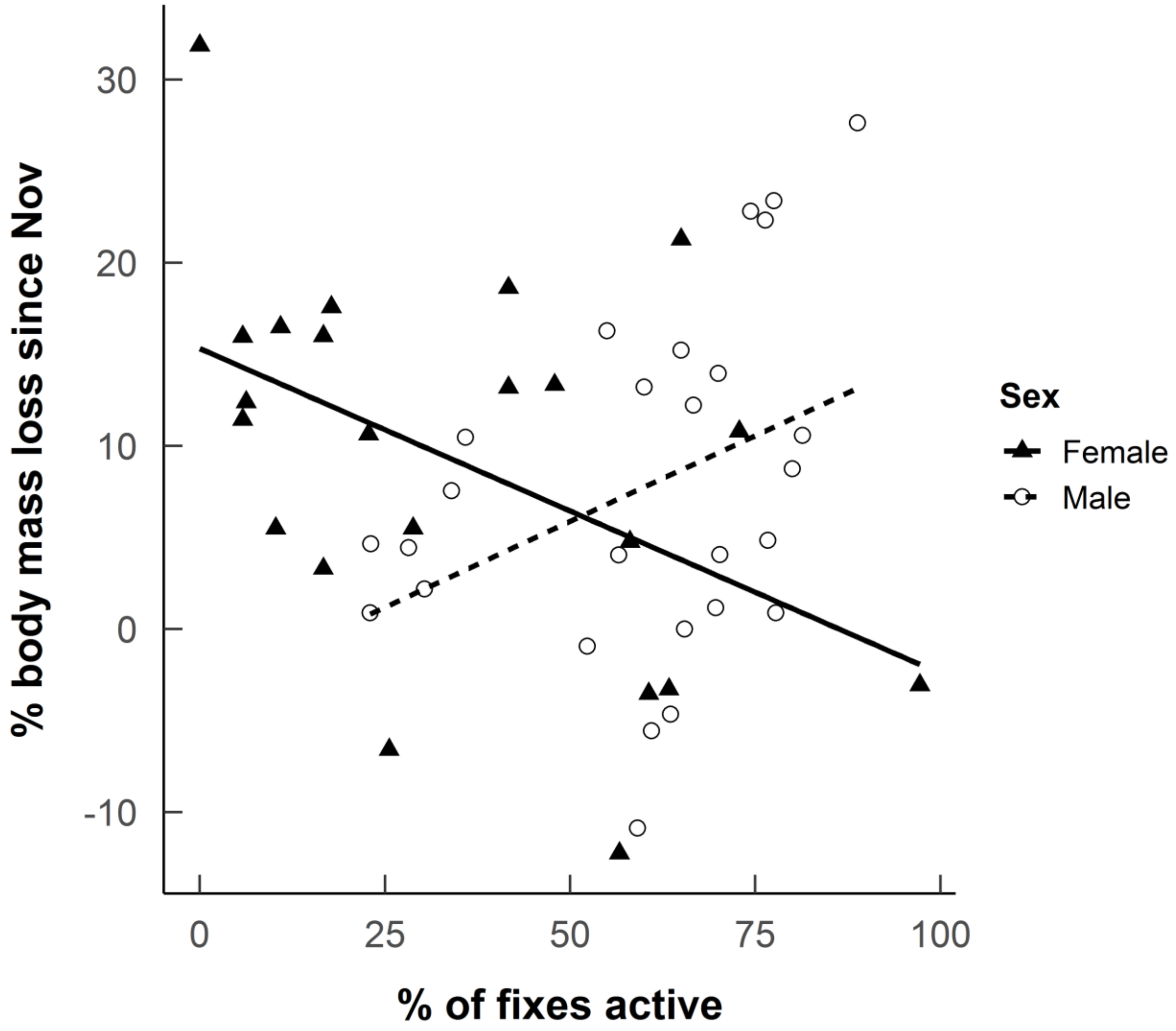

Figure S7. The relationship between a hedgehog's activity that month (measured as the percentage of fixes active and outside of the nest) and the percentage body mass loss of that hedgehog since November (measured in December, January and February). In males the higher the activity the greater the loss in body mass, yet in females the higher activity the lower the body mass loss

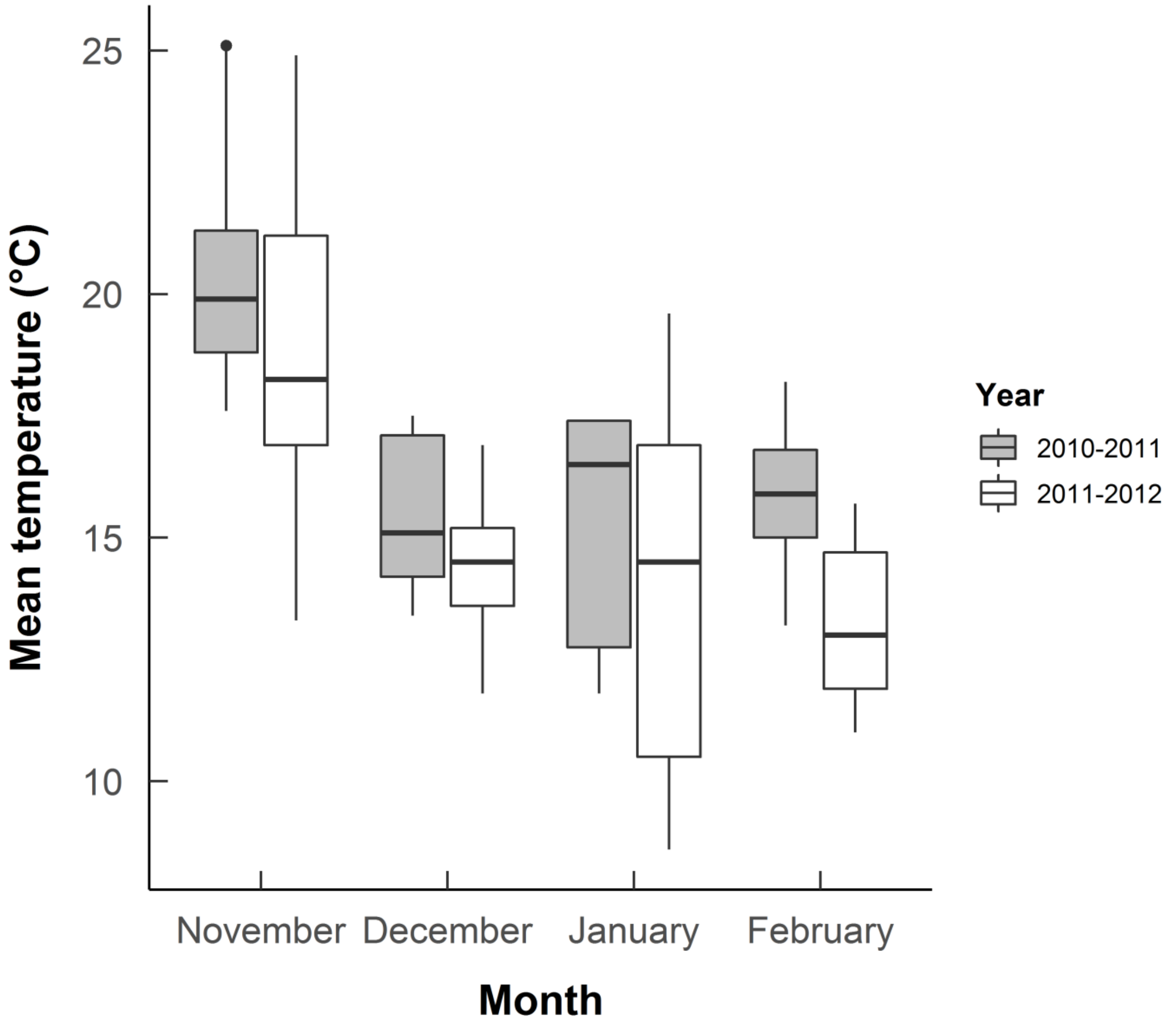

Figure S8. The mean ambient temperature at the study site in Qatar measured whilst recording nightly activity of 20 radio-tagged hedgehogs over winter in 2010-2011 and 2011-2012. Lower and upper hinges show the interquartile range. Whiskers show values within 1.5 times the interquartile range, dots are outliers to this.

Tables

Table S1. Output from a mixed effects model to test factors affecting the percentage of nights a hedgehog spent inactive and inside the nest over winter in Qatar. The percentage was calculated by dividing the number of nights inactive by the total number of nights tracked

**Variable**	**df**	**Chisq**	**P value**	**Effect size**	**95% CI**
Sex (*MALE*)	1	73.103	<0.0001***	-1.555	-1.940, -1.184‡
Year (2011-2012)	1	3.427	0.064.	0.504	-0.030, 1.042
Body mass in Nov	1	0.003	0.958	-0.0001	-0.005, 0.005

The reference categories were: Sex- Female; Year- 2010-2011

.= near significance; *P<0.05; **P<0.01; ***P<0.001

‡95% confidence interval of the effect size does not contain zero

Table S2. Output from a mixed effects model to test factors affecting a hedgehog's activity status on a given night, i.e. whether or not a hedgehog was inactive and in the nest during a night of radio-tracking during winter in Qatar

**Variable**	**df**	**Chisq**	**P value**	**Effect size**	**95% CI**
Sex (*MALE*)	1	75.957	<0.0001***	-6.953	-9.103, -4.900‡
Mean Temperature	1	54.830	<0.0001***	-0.369	-0.472, -0.277‡
Sex* Mean Temp (*MALE*)	1	28.363	<0.0001***	0.342	0.214, 0.474‡

The reference categories were: Sex- Female; Sex*Average Temperature- Female

*P<0.05; **P<0.01; ***P<0.001

‡95% confidence interval of the effect size does not contain zero

Table S3. Output from a mixed effects model to test factors affecting activity of hedgehogs over winter in Qatar. Activity level was measured by radio-tracking and calculated as total hours active outside of nest/number of hours monitored each month

**Variable**	**df**	**Chisq**	**P value**	**Effect size**
Sex (*MALE*)	1	20.018	<0.0001***	1.881
Month *(NOV)*	3	369.937	<0.0001***	1.522
Month (*JAN)*				1.218
Month (*FEB)*				2.475
Sex*Month (*MALE NOV)*	3	283.440	<0.0001***	-1.980
Sex*Month (*MALE JAN*)				-0.084
Sex*Month (*MALE FEB*)				-1.089
Average Temperature	1	1.132	0.2873	0.081

The reference categories were: Sex- Female; Month- Dec; Sex*Month- Male Dec

*P<0.05; **P<0.01; ***P<0.001

Table S3b. Post-hoc tests performed on the significant interaction between sex and month on activity (as displayed above in table S5). Post-hoc tests were performed using the Kenward-Roger degrees of freedom correction

Contrast	Month	Estimate	SE	Z ratio	P value
Female - Male	Nov	0.0994	0.183	0.543	0.5869
Female - Male	Dec	-1.8807	0.209	-8.98	<.0001***
Female - Male	Jan	-1.7965	0.198	-9.078	<.0001***
Female - Male	Feb	-0.7914	0.209	-3.782	0.0002**

Results are given on the log odds ratio (not the response) scale
*P<0.05; **P<0.01; ***P<0.001

Table S4. Output from a mixed effects model to test factors affecting body mass in desert hedgehogs throughout the year in Qatar

Variable	df	F statistic	P value	Effect size	95% CI
Sex (*MALE*)	1, 136	4.164	0.043*	65.766	39.603, 92.239‡
Month (*FEB)*	11,499	23.755	<0.0001***	2.630	-17.252, 22.613
Month (*MAR)*				4.650	-14.507, 23.972
Month (*APR)*				-8.704	-28.955, 11.749
Month (*MAY)*				-3.072	-25.167, 19.063
Month (*JUN)*				5.154	-16.408, 26.770
Month (*JUL)*				22.144	-4.726, 49.112
Month (*AUG)*				-2.875	-28.174, 22.450
Month (*SEP)*				11.399	-17.448, 40.211
Month (*OCT)*				35.613	13.230, 57.945‡
Month (*NOV)*				47.742	23.368, 72.009‡
Month (*DEC)*				11.639	-16.881, 40.076
Sex*Month (*MALE FEB*)	11, 498	8.220	<0.0001***	-65.812	-89.247, -42.550‡
Sex*Month (*MALE MAR)*				-84.497	-107.478, -61.752‡
Sex*Month (*MALE APR)*				-65.772	-89.805, -42.011‡
Sex*Month (*MALE MAY)*				-50.920	-77.007 -24.951‡
Sex*Month (*MALE JUN)*				-61.370	-87.013, -35.876‡
Sex*Month (*MALE JUL)*				-83.947	-116.881, -51.191‡
Sex*Month (*MALE AUG)*				-52.282	-85.260, -19.414‡
Sex*Month (*MALE SEP)*				-33.463	-67.179, 0.194
Sex*Month (*MALE OCT)*				-19.085	-45.499, 7.260
Sex*Month (*MALE NOV)*				-28.819	-57.268, -0.333‡
Sex*Month (*MALE DEC)*				2.599	-29.265, 34.5177
Year (*2011-2012*)	1, 544	9.364	0.002*	-10.576	-17.196, -3.910‡

The reference categories were: Sex- Female; Month- Jan; Sex*Month- Male Jan; Year, 2010-2011
*P<0.05; **P<0.01; ***P<0.001
‡95% confidence interval of the effect size does not contain zero

Table S4b. Post-hoc tests performed on the significant effect of an interaction between sex and month on hedgehog body mass (as displayed above in table S1). Post-hoc tests were performed using the Kenward-Roger degrees of freedom correction

**Contrast**	**Month**	**estimate**	**SE**	**df**	**t.ratio**	**p.value**
Female-Male	January	-65.7662	13.6	376	-4.835	<.0001***
Female-Male	February	0.04592	12.2	272	0.004	0.997
Female-Male	March	18.73093	11.7	247	1.594	0.1122
Female-Male	April	0.00619	11.7	244	0.001	0.9996
Female-Male	May	-14.8461	13.1	335	-1.132	0.2586
Female-Male	June	-4.39598	13.1	339	-0.337	0.7365
Female-Male	July	18.18125	17.1	547	1.062	0.2885
Female-Male	August	-13.4839	17.3	560	-0.78	0.4354
Female-Male	September	-32.3031	17.6	566	-1.838	0.0666
Female-Male	October	-46.6811	13.4	356	-3.479	0.0006***
Female-Male	November	-36.9472	15.2	472	-2.428	0.0155*
Female-Male	December	-68.3651	16.9	541	-4.036	0.0001***

*P<0.05; **P<0.01; ***P<0.001

Table S5. Output from a mixed effects model to test factors affecting the monthly percentage body mass loss of hedgehogs over winter in Qatar. Starting body mass was measured in November

**Variable**	**df**	**F statistic**	**P value**	**Effect size**	**95% CI**
Sex (*MALE*)	1, 28	0.508	0.482	-7.824	-14.839, -0.868‡
Month (*JAN)*	2, 52	8.864	0.0005***	0.211	-6.554, 6.739
Month (*FEB)*				-1.155	-7.832, 5.266
Sex*Month (*MALE JAN*)	2, 52	12.871	<0.0001***	2.147	-5.344, 9.949
Sex*Month (*MALE FEB*)				16.192	8.882, 23.804‡
Year (*2011-2012*)	1, 71	0.033	0.856	-0.182	-3.893, 3.223
Body mass in Nov	1, 40	1.560	0.219	1.249	-0.012, 0.059

The reference categories were: Sex- Female; Month- Dec; Sex*Month- Male Dec; Year- 2010-2011
*P<0.05; **P<0.01; ***P<0.001
‡95% confidence interval of the effect size does not contain zero

Table S5b. Post-hoc tests performed on the significant interaction between sex and month on percentage body mass loss (as displayed above in table S2). Post-hoc tests were performed using the Kenward-Roger degrees of freedom correction

**Contrast**	**Month**	**Estimate**	**SE**	**df**	**T ratio**	**P value**
Female - Male	Dec	7.82	3.69	74.7	2.12	0.0374*
Female - Male	Jan	5.68	3.06	60.1	1.857	0.0683**.**
Female - Male	Feb	-8.37	2.89	56.2	-2.898	0.0053**

.= near significance; *P<0.05; **P<0.01; ***P<0.001

Table S6. Output from a mixed effects model to test factors affecting monthly percentage body mass loss in hedgehogs over winter in Qatar, including activity data from 18 hedgehogs. Starting body mass was measured in November

**Variable**	**df**	**F statistic**	**P value**	**Effect size**	**95% CI**
Sex (*MALE*)	1, 19	0.478	0.497	2.680	-5.015, 10.545
Month (*JAN)*	2, 24	16.770	<0.0001***	-9.112	-15.395, -2.791‡
Month (*FEB)*				-10.530	-17.946, -3.057‡
Sex*Month (*MALE JAN*)	2, 24	16.744	<0.0001***	-8.579	-15.067, -1.927‡
Sex*Month (*MALE FEB*)				8.317	2.037, 14.730‡
Year (*2011-2012*)	1, 33	3.381	0.075.	11.265	0.445, 22.052‡
Body mass in Nov	1, 26	1.590	0.218	0.0490	-0.0214, 0.118
Activity	1, 29	3.589	0.068.	-0.099	-0.193, -0.007
Temperature	1, 24	9.485	0.005**	9.589	3.962, 15.067‡
Humidity	1, 24	16.744	<0.0001***	-1.354	-1.839, -0.859‡

The reference categories were: Sex- Female; Month- Dec; Sex*Month- Male Dec; Year- 2010-2011
.= near significance,*P<0.05; **P<0.01; ***P<0.001
‡95% confidence interval of the effect size does not contain zero

Table S6b. Post-hoc tests performed on the significant interaction between sex and month on percentage body mass loss (as displayed above in table S6). Post-hoc tests were performed using the Kenward-Roger degrees of freedom correction

**Contrast**	**Month**	**Estimate**	**SE**	**df**	**T ratio**	**P value**
Female - Male	Dec	-2.68	4.36	29.7	-0.614	0.5436
Female - Male	Jan	5.9	4.35	28.7	1.355	0.1859
Female - Male	Feb	-11	4.03	24	-2.728	0.0117**

*P<0.05; **P<0.01; ***P<0.001

Fieldwork diary excerpt

From the fieldwork diary of one of the authors (NY) 11th January 2012:

> *The signal of a female adult 116 stopped moving and became "inactive" in the middle of nowhere, so I went to check it. She was courted by two males. One of the males had wet genitalia with some bubbles around the penis – does this mean he successfully copulated with 116? The other male's (smaller than the first male) genitalia was not wet nor there were bubbles. She was courted by another male towards the end of the night up until 05:00 when I "accidentally" intervened the liaison. Then, I checked the radio-tracking records of FA116 on 11th January. Probably she was courted between c. 19:00 – 22:00 by more than two males as the signal was from the location where hedgehogs usually were not stationary. Then, between c. 03:00 and c. 05:00 she may have been courted by at least one male. On that night, she came out of the nest at c. 18:00 and went back to the nest after 06:00. So, possibly five hours out of 12 hours she may not have been able to forage due to males' harassment (e.g. trying to mate her).*
